# Proteomic analysis of infected primary human leucocytes revealed PSTK as potential treatment-monitoring marker for active and latent tuberculosis

**DOI:** 10.1371/journal.pone.0231834

**Published:** 2020-04-16

**Authors:** Benjawan Kaewseekhao, Sittiruk Roytrakul, Yodying Yingchutrakul, Kanin Salao, Wipa Reechaipichitkul, Kiatichai Faksri

**Affiliations:** 1 Department of Microbiology, Faculty of Medicine, Khon Kaen University, Khon Kaen, Thailand; 2 Research and Diagnostic Center for Emerging Infectious Diseases (RCEID), Faculty of Medicine, Khon Kaen University, Khon Kaen, Thailand; 3 National Center for Genetic Engineering and Biotechnology (BIOTEC), National Science and Technology Development Agency (NSTDA), Pathumthani, Thailand; 4 Department of Medicine, Faculty of Medicine, Khon Kaen University, Khon Kaen, Thailand; Jamia Hamdard, INDIA

## Abstract

Markers for monitoring clearance of *Mycobacterium tuberculosis* (*Mtb*) infection during anti-TB drug treatment could facilitate management of tuberculosis (TB) treatment, but are lacking. We aimed to screen for *Mtb* clearance markers from *in-vitro*-infected leucocytes and to evaluate these markers in followed-up active TB (ATB) patients and latent TB (LTBI) cases after anti-TB drug treatment. Extracellular proteins from primary leucocytes infected with each of the *Mtb* lineages (East-Asian, Indo-Oceanic, Euro-American and the laboratory strain H37Rv) were screened as possible clearance markers. Leucocytes infected with *Staphylococcus aureus* acted as controls. The proteomic analysis was performed using GeLC-MS/MS. Several quantitative and qualitative candidate clearance markers were found. These proteins were suppressed during the infection stage of all *Mtb* lineages and re-expressed after bacillary clearance. PSTK, FKBP8 and MGMT were common clearance markers among the four *Mtb* lineages in our model. Only PSTK was a potential clearance marker based on western blot validation analysis from culture supernatants. The PSTK marker was further validated with western blot analysis using serum samples (n = 6) from ATB patients and LTBI cases during anti-TB drug treatment, and from healthy controls (n = 3). Time-dependent increase of PSTK was found both in ATB and LTBI patients during the course of anti-TB drug treatment, but not in healthy controls. We have demonstrated that PSTK is a potential treatment-monitoring marker for active and latent TB.

## Introduction

Annually, around 1.6 million tuberculosis (TB) deaths and 10 million new cases are reported [1]. One-third of the world’s population is assumed to have latent TB infection (LTBI) and 5–10% of them can progress to active TB (ATB) [2]. ATB is treated with anti-TB drugs for 6–9 months (2 months of isoniazid, rifampicin, pyrazinamide and ethambutol (2IRZE) followed by 4 months of isoniazid and rifampicin (4IR)) and LTBI is treated with isoniazid for 6–9 months [3]. The global TB treatment success rate is 85% for drug-susceptible TB, 56% for multidrug-resistant TB (MDR-TB) and 39% for extensively drug-resistant TB (XDR-TB) [1]. The rate of relapse of TB, indicating treatment failure, is high, ranging from 4.7% to 50% [4–7]. Proportion of multidrug-resistant TB is also high (18%) in previously treated cases [1]. Tools to increase the treatment success rate are crucially needed to accomplish the goals of the World Health Organization End-TB program by 2035 [1]. The lack of an effective marker to indicate clearance of TB hinders successful treatment. This is highly relevant in the case of LTBI for which adjustment of treatment regimen and duration is needed to ensure bacillary clearance, something that cannot determined by classical markers such as acid-fast bacilli staining.

The clearance of *Mtb* from TB patients is typically assumed on the basis of clinical and radiological improvement supplemented by sputum microscopy and/or *Mtb* culture. However, conventional markers for TB treatment monitoring, such as acid-fast bacilli staining and nucleic-acid detection, are not sensitive enough to indicate complete clearance of *Mtb* from the host [8]. This has prompted a continuing search for novel biomarkers for monitoring treatment of TB and eventual clearance of bacilli. Markers previously proposed to indicate a decrease of *Mtb* burden in a host include IFN-γ levels in sera of TB patients [9], MMP-8 [10] and cytokine profiles [11,12]. However, none of them was sensitive enough to indicate complete elimination of *Mtb* infection from the tissues.

Previously, our group reported a preliminary study exploring potential clearance markers using a monocytic cell line (THP-1 cells) [13]. However, testing using a wider range of *Mtb* lineages and other bacteria as controls is required, as well as clinical sample validation.

In this study, we investigated clearance biomarkers from *Mtb*-infected leucocytes treated with isoniazid (INH)/ rifampicin (RIF), analyzed by LC-MS/MS and further validated using prospectively collected clinical samples from TB patients undergoing treatment for ATB and LTBI. The clearance biomarkers we identified have potential use for treatment monitoring and determining the success of TB treatment in both ATB and LTBI.

## Methods

### Study design and setting

**Fig 1:**
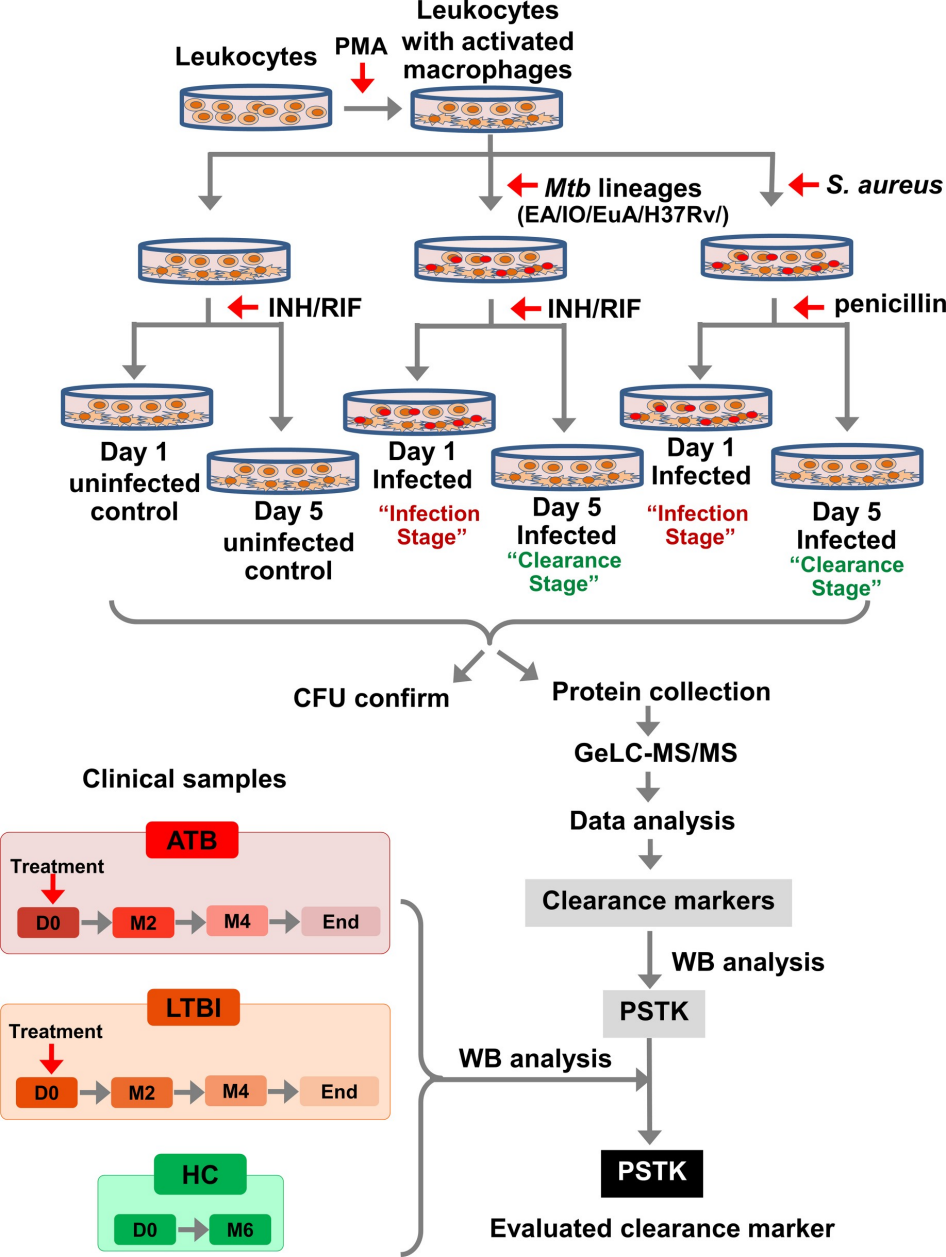
Study design and experiment workflow. The study was divided into two parts, the *in-vitro* screening of clearance biomarkers using GeLC-MS/MS (upper part) and the clinical sample validation of the candidate clearance biomarker (PSTK) using western blot analysis. INH = isoniazid, RIF = rifampicin and INH/RIF refers to 3: 9 μg/ml of INH: RIF drug treatment.

To identify clearance biomarkers for *Mtb*, primary leucocytes, cells from three healthy donors were used as the *in-vitro* host model (Fig 1). Individual lines of these cells were infected with three lineages of *Mtb* (East-Asian (EA), Indo-Oceanic (IO) and Euro-American (EuA)) commonly found in Southeast Asia [14]. A fourth cell line was infected with the laboratory control strain *Mtb* H37Rv. Cells infected with *Staphylococcus aureus* ATCC 25923 were used as non-*Mtb* controls. Our criteria for identifying potential clearance markers were that they should be molecules found after clearance of all *Mtb* lineages but not found in cells currently infected with *Mtb* or in uninfected controls. Nor should such markers be found in cells infected with, or cleared of, *S*. *aureus*. The colony-forming unit (CFU) count assay on Middlebrook (M) 7H11 media was used to assess the *in-vitro* clearance stage. GeLC-MS/MSI analysis was used for the screening of leukocyte culture supernatant to identify potential biomarkers. Western blot analysis was used to validate the clearance marker received from the GeLC-MS/MSI. Validation of the candidate clearance marker using serum samples from ATB and LTBI cases before, during and after completion of treatment was performed using western blot analysis. Blood samples from healthy controls at day 1 and subsequently collected 6 months later were used as negative controls.

### Bacterial culture and inoculum preparation

Three of the tested *Mtb* strains (EA, IO and EuA) were isolated from clinical samples and were pan-susceptible to all anti-TB drugs. The laboratory control strain, *Mtb* H37Rv, was also pan-susceptible. All *Mtb* strains were cultured in Middlebrook 7H9 with OADC supplements for 14 days. The concentrations of *Mtb* cells were measured and adjusted to 0.5 McFarland standards. Clumps in *Mtb* cell suspensions were removed by passing through a 26-gauge needle. *Staphylococcus aureus* (ATCC25923) was cultured at 37°C in Luria Broth (LB) medium for 24 hours.

### Participants and sample collection

Patients with ATB had clinical symptoms of TB and at least one positive form of microbiological evidence; staining of acid-fast bacilli, bacterial culture or a molecular test (Xpert MTB/RIF, Cepheid, Sunnyvale, CA, USA). Individuals with LTBI were healthcare workers exposed to TB with a QFT-positive result. HC refers to healthy persons with no evidence of TB exposure. ATB cases infected with drug-sensitive *Mtb* were treated with standard regimens (2IRZE and 4IR). LTBI cases were treated with standard prophylactic treatments (isoniazid for 9 months). After recruitment, any participant who developed active TB or diabetes mellitus was excluded from analysis. Any ATB participant who developed drug-resistant TB was excluded from analysis. Serum samples of the TB cases (ATB = 3 and LTBI = 3) were collected before, during (2 and 4 months after the start of treatment) and after completion of treatment (6 or 9 months). Serum samples from healthy controls (HC = 3) were collected at the start point (first day of recruitment) and six months after recruitment. The three ATB patients completed treatment in six (n = 1) or nine (n = 2) months. All three LTBI cases completed the treatment within nine months. Consent forms were obtained from all participants. Demographic data of the participants are described in S1 Table. None of the participants was a minor: the youngest was 24 years old. This study protocol was approved by Human Research Ethics, KKU with protocol number HE581377.

## Isolation of human leukocytes

Venous blood samples from three healthy participants were collected. Primary leukocytes were isolated using the HetaSep protocol (StemCell Technologies, Vancouver, Canada) [15]. Isolated cells were cultured using RPMI medium. Monocytes were activated by incubation with 50 nM phorbol myristate acetate (PMA) for 24 hours.

### Leukocyte infection and *in-vitro* clearance assay

The activated cells were infected with four *Mtb* strains (EA, IO, EuA and H37Rv) and incubated for 4 hours. Then, the infected cells were treated with a combination of isoniazid (INH) and rifampicin (RIF) with 3: 9 μg/ml of INH:RIF (optimized concentrations according to a previous study [13] for intracellular bacillary killing within three days). Medium containing the drugs was replaced in cell cultures every 24 hours for up to five days. The extracellular proteins were collected from infected cells 24 hours after the start of treatment (infection stage) and five days after the start of treatment (clearance stage). Cells infected with *S*. *aureus* were treated with an optimized concentration of penicillin (4 μg/ml) for intracellular bacterial killing within three days. Uninfected cells treated with the combination of INH and RIF were used as negative controls. CFU counts were done by dropping the culture cell pellets and supernatant onto M7H11 plates and incubating these for at least four weeks to unsure the *in-vitro* clearance of *Mtb* (S2 Table).

### Extracellular protein collection and preparation

The culture supernatant (three ml) from each well was collected and mixed with SDS (final concentration of 0.5% w/v). Protein concentrations were measured using the Lowry method [16]. Five microliters of BSA were used as standard (0, 2, 4, 6, 8, 10 μg) and five μl of each sample were transferred into 96-well plates (in triplicate). Each sample was incubated with 200 μl of solution A (2.5% SDS, 2.5% Na_2_CO_3_, 0.2 N NaOH, 0.025% CuSO_4_ and 0.05% tartaric acid) at room temperature for 30 minutes. Then the plates were incubated for 30 minutes at room temperature with 50 μl of solution B (20% Folin-Ciocalteu phenol reagent). The optical density of each sample was measured at 750 nm and the concentration was calculated by comparison to the standards.

### SDS PAGE and in-gel digestion

In-gel digestion of protein samples was done to exclude the bovine serum albumin and to ensure equal initial protein concentrations free of agents that might interfere with optical measuring such as INH and RIF. The protein samples were separated by SDS-PAGE (50 μg of each sample). Protein-containing gels were stained with Coomassie blue. The gel in each well was carefully sliced into 11 pieces and each gel plug was further cut into 1-mm^3^ cubes. The gel was transferred into a 96 well plate and tryptic digestion was performed. The gel pieces were incubated for 10 minutes with 25 mM NH_4_HCO_3_ then for 10 minutes with 200 μl acetonitrile (ACN). Ten mM DTT in 10 mM NH4HCO3 was added after ACN removal and the plate incubated at 56°C for one hour. Next, 100 mM iodoacetamide in 10 mM NH_4_HCO_3_ was added and the gel pieces were incubated at room temperature for one hour in the dark. Liquid was removed using two washes with 200 μl of ACN followed by removal of all liquid. Tryptic digestion was performed by incubation of the gel pieces at 37°C for three hours with enzyme solution (10 ng/μl trypsin in 10 mM NH_4_HCO_3_). For protein extraction, 50% ACN was added thrice to the gel pieces and shaken at room temperature for 10 minutes. Peptide solutions were transferred into new low-protein-binding 96-well plates and dried at 40°C. The dry peptides were kept at -20°C until analyzed.

### LC-MS/MS analysis

The peptide samples were suspended in 10 μl of 0.1% formic acid and transferred into low-protein-binding tubes. The samples were centrifuged at 8,000 g for 10 minutes and transferred into new tubes. Then, 4.5 μl of each peptide sample was injected into a LC-MS/MS analyzer (Hybrid quadrupole Q-TOF impact II^™^, Bruker Daltonics, United States). Separation of tryptic peptides was performed using the Ultimate3000 Nano/Capillary LC System (Thermo Scientific, UK) coupled with Nano-captive spray ion source. Mobile phase A (0.1% formic acid) was added into the column to transfer the samples with flow rate of 15 μl/minute for one minute. Mobile phase B (5–50% solution of 0.1% formic acid in 80% acetonitrile) was added for peptide separation with a flow rate of 600 nl/ minute for 15 minutes. Electrospray ionization was carried out at 1.6 kV using the CaptiveSpray. Then, the column was rinsed thrice with 80% mobile phase B at 35°C. The MS/MS survey was set to separate proteins ranging from 150 to 3,000 Da with 0.5 sec scan time.

### Western blot analysis

Extracellular proteins from culture supernatant and serum samples of patients and controls were used for validation of biomarkers. Protein samples (50 μg) were separated by 12.5% SDS-PAGE and proteins transferred onto nitrocellulose membranes (0.2 μm, Bio-Rad, USA) using a blotting machine (Trans-Blot SD semi-dry transfer cell, Bio-Rad, USA) at 23 V for 30 minutes. The protein-containing membranes were washed twice with TBS-Tween solution for five minutes. The blotted membranes were blocked using 5% BSA in a TBS-Tween and incubated at 4°C overnight. Protein detection was performed by adding primary antibody solution (mouse anti-PSTK at 1:100 dilution (Santa Cruz Biotechnology, USA), mouse anti-FKBP8 at 1:100 dilution (SANTA CRUZ, USA), and mouse anti-transferrin at 1:2,000 dilution (SANTA CRUZ, USA)) to the blotted membranes. The membranes were incubated at room temperature for three hours and then washed thrice for five minutes with a TBS-Tween solution. The secondary antibody solution (HRP-conjugated anti-mouse IgG (SANTA CRUZ, USA) at 1:2,000 dilution) was added to the blots and incubated for two hours and washed thrice with a TBS-Tween solution. Chemiluminescence signals were enhanced using chemiluminescent reagents (Thermo Scientific, USA) and were analyzed using Image Quant LAS 4000 software (GE Healthcare Life Science, UK). Western blot signal intensity was analyzed using ImageJ [17].

### Bioinformatics and data analyses

LC-MS/MS data were analyzed using DeCyderMS 2.0 differential analysis software (DeCyderMS, GE Healthcare Life Science, UK). The output from DeCyderMS was submitted for a database search using Mascot software (Matrix Science, London, UK) and protein identification based on an NCBI database search. The following search parameters were used: taxonomy (human or eukaryote), enzyme (trypsin), variable modifications (carbamidomethyl, oxidation of methionine residues), mass values (monoisotopic), protein mass (unrestricted), peptide mass tolerance (± 1.2 Da), fragment mass tolerance (± 0.6 Da), peptide charge state (1+, 2+, and 3+), and max missed cleavages. Construction of a Venn diagram of the biomarker subset among groups was done using VennDiagram packages in the R programming language.

Potential clearance markers were classified into two groups, (i) qualitative markers based on highly stringent screening criteria, i.e. biomarkers only detected from the clearance stage but not detected in the infection controls, uninfected control or *S*. *aureus* controls (ii) quantitative markers based on relative quantity criteria, i.e. biomarkers that were detected in at least one strain of *Mtb*, not detected in *S*. *aureus* controls, and highly expressed (at least four-fold higher than in uninfected controls).

## Results

### Potential *Mtb* clearance markers identified from comparative proteomics of culture supernatant

**Fig 2:**
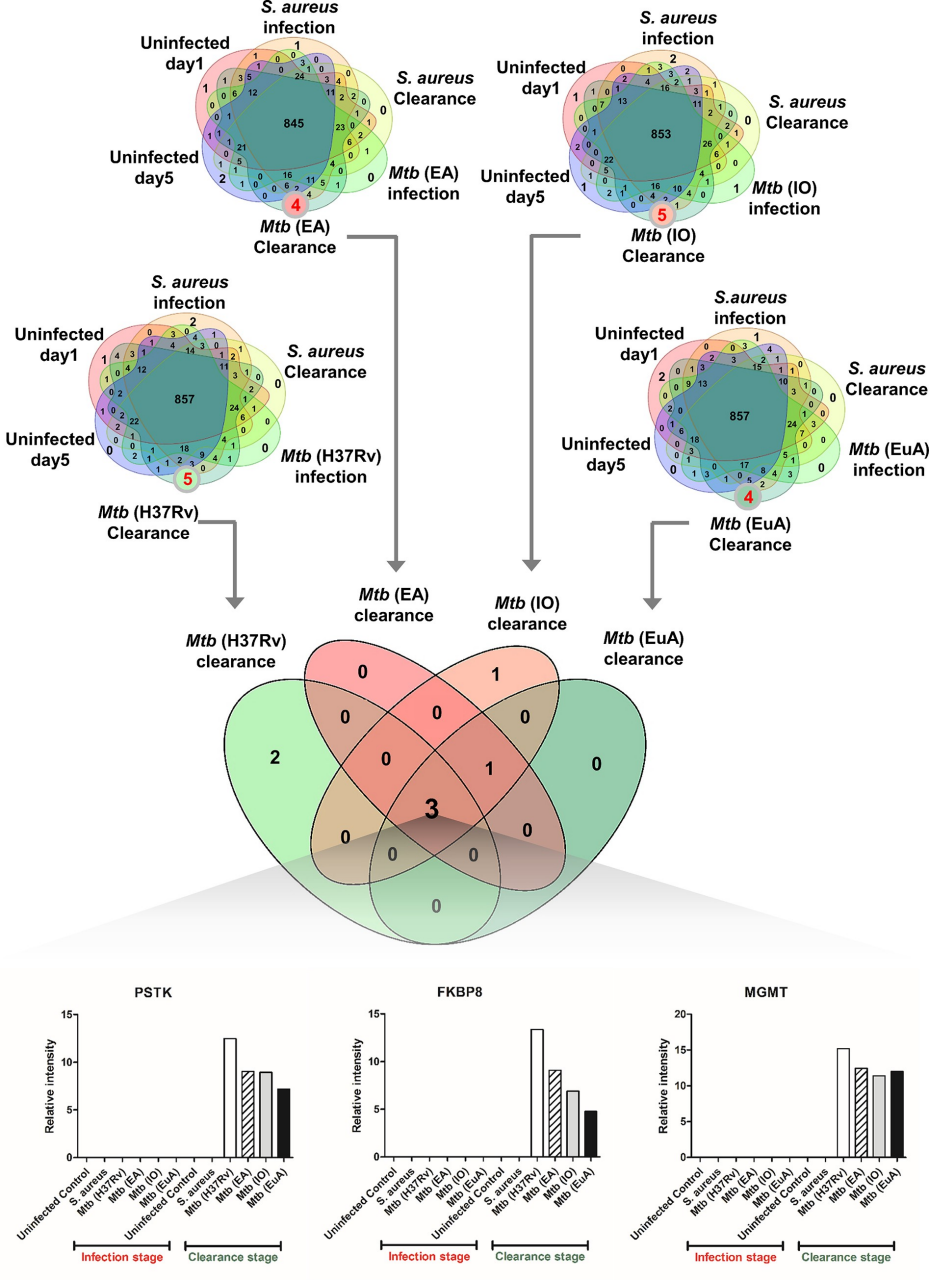
Lineage-independent clearance biomarkers selected from LC-MS/MS analysis. Venn diagram of the proteome showing the number of unique peptides detected in each condition. Three clearance markers (PSTK, FKBP8 and MGMT) were commonly detected in four *Mtb* strains including H37Rv, East-Asian, Indo-Oceanic (IO), Euro-American (EuA) but not detected in the infection stage, background metabolism (normal uninfected cells) and *S*. *aureus*-infected controls. The protein intensities from LC-MS/MS analysis are shown in boxplots. The biomarker screening was performed in three independent experiments and pooled samples submitted for LC-MS/MS analysis.

LC-MS/MS data analysis of culture supernatants revealed three potential TB clearance biomarkers; PSTK (Phosphoseryl-tRNA kinase), FKBP8 (Peptidyl-prolyl cis-trans isomerase FKBP8) and MGMT (O6-methylguanine-DNA methyltransferase). Based on highly stringent criteria, these biomarkers were uniquely and commonly detected in the clearance stages of all four *Mtb* strains (EA, IO, EuA and H37Rv) but were not detected in the infection stage, background metabolism (normal uninfected cells) or *S*. *aureus*-infected controls (Fig 2). An additional set of 15 clearance biomarkers was identified using less stringent criteria (detected only in particular strains of *M*. *tuberculosis* at four-fold higher levels than during the infection stage and not detected in uninfected controls and *S*. *aureus* controls) (S1 Fig). A list of all clearance biomarkers is given in S3 Table.

### Validation of the candidate biomarkers identified from culture supernatants using western blot analysis

**Fig 3:**
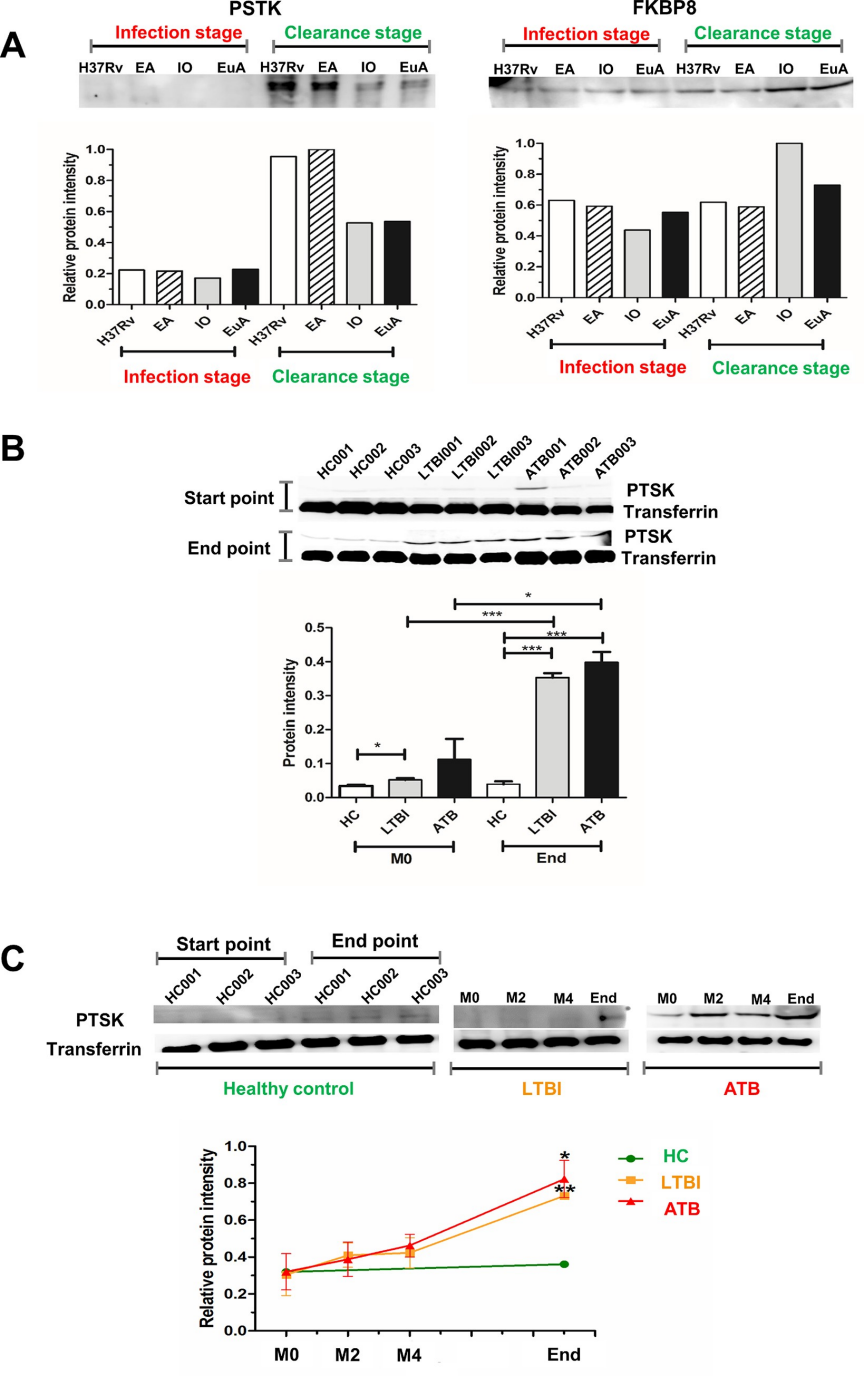
Clearance biomarker validation using western blot analysis. Western blot analysis of PSTK and FKBP8 in extracellular protein (A). Western blot analysis of PSTK in serum samples compared between start point (before treatment) and end point (treatment completion) of ATB, LTBI and HC samples (B). Western blot analysis of PSTK in serum samples compared among samples taken before treatment (M0), after treatment 2 months (M2), 4 months (M4) and 6–9 months (End) with anti-tuberculosis drugs for ATB and LTBI. In healthy controls (HC), the start point (first day of recruitment) and end point (six months after recruitment) were used as negative controls) (C). ATB = active tuberculosis patients, LTBI = latent tuberculosis infections; HC = healthy controls. * = P-value <0.05, ** = P-value <0.01; *** = P-value <0.001.

Two extracellular proteins (PSTK and FKBP8) were screened using western blot analysis to determine if they could be detected in serum. It was found that only PSTK showed an increased level in the clearance stage compared to the infection stage (Fig 3A). Raw figures of blotted gel images are shown (S2 Fig).

### Evaluation of candidate clearance biomarkers using serum from ATB patients and LTBI cases after anti-TB treatment

PSTK was selected to be further validated in serum samples of ATB (n = 3) and LTBI (n = 3) cases during anti-TB drug treatment and HC (n = 3) were used as controls. PSTK levels at completion of drug treatment were significantly higher than before starting the treatment or compared with HC in both ATB (P<0.001) and LTBI (P<0.0001) cases (Fig 3B). The PSTK protein level was significantly correlated with duration of treatment (0 and 2, 4 and 6 or 9 months after the start of drug treatment) in a time-dependent manner in both ATB (P<0.0001) and LTBI (P<0.01) cases (Fig 3C).

## Discussion

The discovery of *Mtb* clearance markers that can be used as TB treatment monitoring markers is difficult in part because of the difficulty of confirming clearance of *Mtb* from the tissues of patients. Previously, we successfully demonstrated that THP-1 cells infected with *Mtb* strain H37Rv could be treated with anti-TB drugs leading to confirmed *in-vitro* clearance of bacilli [13]. With a simple cell-line model, we demonstrated that SSFA2, NCOR2, MCM2, RANBP1, RPTN, ELFN1, RFC2, SETX and CEACAM18 were potential clearance biomarkers [13]. In the current study, we used a more complex model with stringent criteria to select candidate clearance biomarkers that could be used clinically. The primary leukocyte cell culture was used to ensure that complex immune cells were the source of any protein released to the serum of the patient and that *in-vitro* clearance of bacilli was achieved. Testing of four strains of *Mtb* (H37Rv, EuA, IO and EuA) allowed us to cover the heterogeneous range of *Mtb* lineages. The markers must not be found in the background metabolism of normal cells (uninfected cells on day 1) or when the cells get older (uninfected cells on day 6), or during the infection stage before the start of treatment. Cells infected with *S*. *aureus* were used as the other bacterial control to ensure the specificity of the clearance markers.

Only three biomarkers, PSTK, FKBP8 and MGMT, that met the highly stringent criteria were found from the high-throughput screening assay. PSTK and FKBP8 are secreted proteins whereas MGMT is an intracellular protein. MGMT might be released from lysed cells during the 24-hour collection period for cell-culture supernatant. As we were seeking a marker that should be found in serum, the intracellular protein, MGMT, was not subjected to further validation. Hence, PSTK and FKBP8 were then validated using western blot analysis. This showed that levels of FKBP8 from the culture supernatant were not substantially different between the infection and the clearance stage. The failure of western blot analysis of FKBP8 to confirm the LC-MS/MS analysis might be due to the different discriminatory power and resolution of detection of the two techniques. Only PSTK was found to be highly secreted in the clearance stage compared to the infection stage and therefore was selected for further clinical validation with sera from several patients. This step confirmed the potential of PSTK as a TB treatment-monitoring marker. Western blot analysis of PSTK at day 0 (before drug treatment) and 2, 4 and 9 months after the start of treatment also confirmed that the level of PSTK was significantly correlated with the duration of treatment. Although we cannot confirm that these three ATB and three LTBI cases achieved complete eradication of *Mtb*, we did not observe recurrent disease in these individuals at follow-up more than 12 months after completion of treatment.

Phosphoseryl-tRNA kinase (PSTK) protein is an intermediate enzyme involved in the synthesis of selenoproteins, associated with selenium levels [18]. PSTK has a protective role in oxidative stress in early lung injury [19], is associated with cell protection from ROS effects [20] and has a role in induced cellular homeostasis and cell-cycle progression [21]. During TB infection, serum selenium levels of TB patients are lower than in healthy controls [22]. In addition, administration of selenium plus vitamin E to ATB patients improves clinical outcomes relative to untreated patients [23]. Concordantly, we found that PSTK levels increased in the clearance stage of *Mtb* from infected leukocytes in cell culture and PSTK levels also increased after treatment completion in both ATB and LTBI cases in a time-dependent manner during the course of treatment: PSTK levels may be associated with successful treatment and clearance of TB in patients.

Western blot analysis showed some gradation in PSTK levels between the test and control groups. Therefore, it is necessary for future research to establish expression-level cutoffs for PSTK appropriate for indicating *Mtb* clearance. Our study included only 3 participants per group to validate the serum PSTK level. Larger sample sizes with clinical data should be used in the future to confirm the validity of PSTK as a clearance marker. Regardless of the exact cutoff level, the PSTK clearance marker might be simply applied by monitoring whether or not the bacillary burden decreased in tissues after treatment. This is crucial for the treatment of MDR and XDR-TB whereby the marker indicating the treatment response is the key determinant of treatment outcome. It is also important for tracking LTBI prophylactic treatment for which no tools or markers are available to indicate treatment response except for the TST/ IGRA conversion tests, both dependent on the long-lasting response of memory T-cells [24]. We used *S*. *aureus* to represent a bacterial pathogen commonly found in human hosts and to ensure that the clearance markers were not also found following clearance of this bacterial species. However, further validation of potential treatment-monitoring markers is required. For example, other respiratory pathogens, especially nontuberculous mycobacteria (NTM) and pulmonary bacterial pathogens, might influence levels of these markers. NTMs do not respond to anti-TB drugs. Therefore NTM colonization or co-infection in TB patients should not affect the response of clearance markers. We included *S*. *aureus* as the non-TB bacterial control and tested responses to various strains of *Mtb* to cover pathogen heterogeneity. Due to our very stringent criteria, it is difficult to identify other bacterial species that could be used as controls. Therefore, it is still uncertain that PSTK will be a highly specific clearance marker for TB. Other bacteria causing mixed infections or colonization that also respond to anti-TB drugs might confound the response of the clearance markers.

## Conclusion

High-throughput proteomic screening using an *in-vitro* clearance model of leukocyte cultures infected with four strains of *Mtb* showed that secreted PTSK significantly increased in the clearance stage compared to controls. Validation was done using clinical specimens from ATB and LTBI cases, which showed a significant increase in levels of PTSK during the course of treatment in a time-dependent manner. This supported the idea that PTSK is a potential biomarker for monitoring of TB treatment.

## Supporting information

S1 Fig Additional clearance biomarkers. An additional set of 15 clearance biomarkers was detected using less stringent criteria (detected only in particular strains of *M*. *tuberculosis* with four-fold higher expression than during the infection stage and not detected in uninfected control or *S*. *aureus* controls). The level of expression is presented as absent in blue, lowest in light pink, and highest in red according to the density key. Gene symbols are labeled for each protein.
(TIF)

S2 Fig All raw blotted gel images from the experiments reported in this study. The raw blotted gel images (right) link to Fig 3 (left).
(TIF)

S1 Table Demographical characteristics of the TB-infection categories. ATB = active tuberculosis patients (symptomatic patients positive for acid-fast bacilli); LTBI = latent tuberculosis infections (TB-exposed persons with IGRA-positive results); HC = healthy controls (healthy persons with no known risk of TB exposure and with IGRA-negative results); IRZE = isoniazid, rifampicin, pyrazinamide and ethambutol combined drug treatment, IR = isoniazid and rifampicin drug treatment; I = isoniazid drug treatment, None = No antibiotic treatment was applied in the HC group. No additional antibiotics were used in any participant group during the anti-TB drug-treatment course.
(DOCX)

S2 Table Colony forming unit (CFU) assays confirming the *in-vitro* clearance stage. Growth = bacterial cell growth in agar plate, NG = no bacterial cell growth.
(DOCX)

S3 Table List of clearance biomarkers. Both qualitative (based on highly stringent criteria) and quantitative (based on less stringent criteria).
(XLSX)
