# Supplementary figures and images for: Proteomic analysis of infected primary human leucocytes revealed PSTK as potential treatment-monitoring marker for active and latent tuberculosis
Source: PLoS One. 2020 Apr 16;15(4):e0231834. doi: 10.1371/journal.pone.0231834 (PMC7162486; doi:10.1371/journal.pone.0231834)

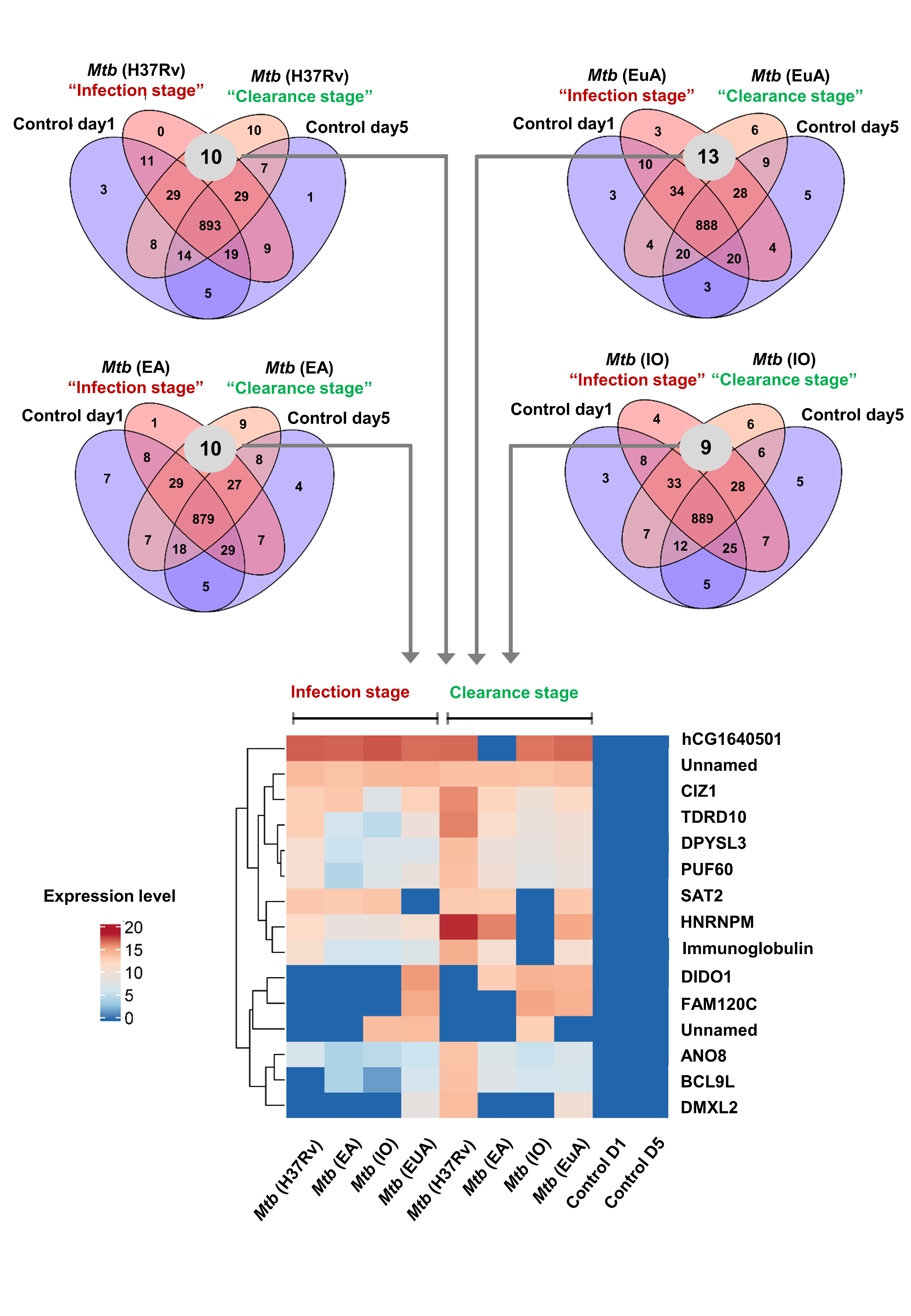

Supplement: S1 Fig — An additional set of 15 clearance biomarkers was detected using less stringent criteria (detected only in particular strains of M. tuberculosis with four-fold higher expression than during the infection stage and not detected in uninfected control or S. aureus controls). The level of expression is presented as absent in blue, lowest in light pink, and highest in red according to the density key. Gene symbols are labeled for each protein. (TIF) [file pone.0231834.s001.tif]

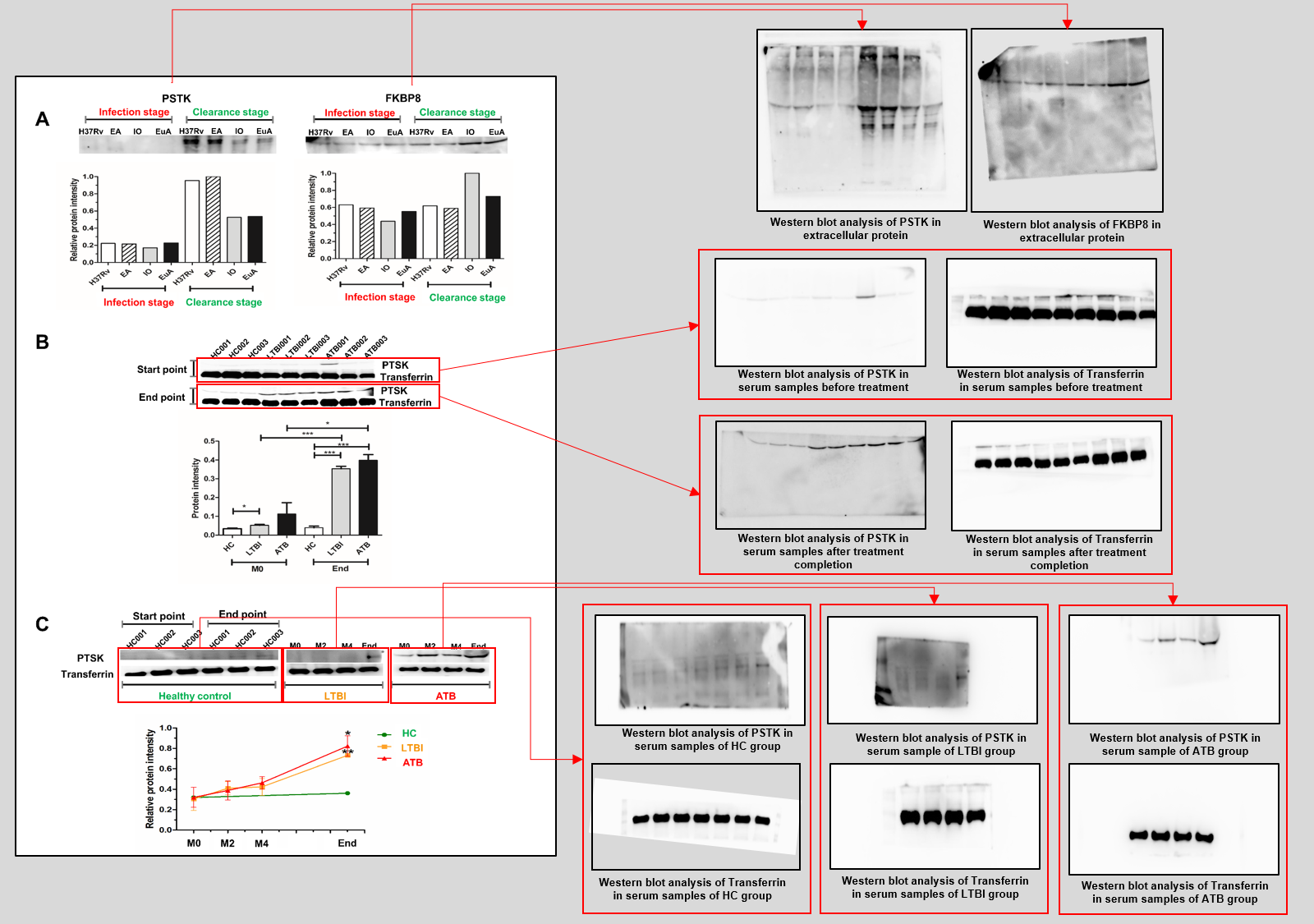

Supplement: S2 Fig — The raw blotted gel images (right) link to Fig 3 (left). (TIF) [file pone.0231834.s002.tif]
