# Supplementary material for: Proteomic analysis of infected primary human leucocytes revealed PSTK as potential treatment-monitoring marker for active and latent tuberculosis
Source: PLoS One. 2020 Apr 16;15(4):e0231834. doi: 10.1371/journal.pone.0231834 (PMC7162486; doi:10.1371/journal.pone.0231834)
Supplement: S1 Table — ATB = active tuberculosis patients (symptomatic patients positive for acid-fast bacilli); LTBI = latent tuberculosis infections (TB-exposed persons with IGRA-positive results); HC = healthy controls (healthy persons with no known risk of TB exposure and with IGRA-negative results); IRZE = isoniazid, rifampicin, pyrazinamide and ethambutol combined drug treatment, IR = isoniazid and rifampicin drug treatment; I = isoniazid drug treatment, None = No antibiotic treatment was applied in the HC group. No additional antibiotics were used in any participant group during the anti-TB drug-treatment course. (DOCX) [file pone.0231834.s003.docx]

**S1 Table.** Demographical characteristics of the TB-infection categories.

| **Characteristics** | **ATB**  **(n = 3)** | **LTBI**  **(n = 3)** | **HC**  **(n = 3)** |
| --- | --- | --- | --- |
|  |  |  |  |
| Age mean ± SD | 45.67 ± 15.01 | 27.3 ± 3.05 | 30.67 ± 3.05 |
| BMI mean ± SD | 21.07 ± 2.45 | 21.53 ± 2.45 | 27.01 ± 8.7 |
| Gender male , n (%) | 2 (67%) | 2 (67%) | 1 (33%) |
| Treatment, month (drug) | 2IRZE + 4IR/ 2IRZE + 7IR | 9 (I) | None |

ATB = active tuberculosis patients (symptomatic patients positive for acid-fast bacilli); LTBI = latent tuberculosis infections (TB-exposed persons with IGRA-positive results); HC = healthy controls (healthy persons with no known risk of TB exposure and with IGRA-negative results); IRZE = isoniazid, rifampicin, pyrazinamide and ethambutol combined drug treatment, IR = isoniazid and rifampicin drug treatment; I = isoniazid drug treatment, None = No antibiotic treatment was applied in the HC group. No additional antibiotics were used in any participant group during the anti-TB drug-treatment course.
