# Supplementary material for: Proteomic analysis of infected primary human leucocytes revealed PSTK as potential treatment-monitoring marker for active and latent tuberculosis
Source: PLoS One. 2020 Apr 16;15(4):e0231834. doi: 10.1371/journal.pone.0231834 (PMC7162486; doi:10.1371/journal.pone.0231834)
Supplement: S2 Table — Growth = bacterial cell growth in agar plate, NG = no bacterial cell growth. (DOCX) [file pone.0231834.s004.docx]

**S2 Table.** Colony forming unit (CFU) assays confirming the *in-vitro* clearance stage.

| Conditions | Day1 | Day3 | Day5 |
| --- | --- | --- | --- |
| Uninfected control | NG | NG | NG |
| *Staphylococcus aureus* | Growth | NG | NG |
| *Mycobacterium tuberculosis* (H37Rv) | Growth | NG | NG |
| *Mycobacterium tuberculosis* (EA) | Growth | NG | NG |
| *Mycobacterium tuberculosis* (IO) | Growth | NG | NG |
| *Mycobacterium tuberculosis* (EUA) | Growth | NG | NG |

Growth= Bacterial cell growth in agar plate, NG=No bacterial cell growth.
